# Supplementary material for: l-Buthionine Sulfoximine Detection and Quantification in Polyurea Dendrimer Nanoformulations
Source: Molecules. 2019 Aug 27;24(17):3111. doi: 10.3390/molecules24173111 (PMC6749587; doi:10.3390/molecules24173111)
Supplement: Supplementary file 1 [file molecules-24-03111-s001.zip › molecules-583732-SI.pdf]

Supporting Information

Pedro Mota,[1] Rita F. Pires,[1] Jacinta Serpa[2,3] and Vasco D. B. Bonifácio[1]

[1]*CQFM-IN and IBB-Institute for Bioengineering and Biosciences, Instituto Superior Técnico, Universidade de Lisboa, 1049-001 Lisboa, Portugal.*

[2]*CEDOC, Chronic Diseases Research Centre, NOVA Medical School, Faculdade de Ciências Médicas, Universidade NOVA de Lisboa, Campo dos Mártires da Pátria, 130, 1169-056 Lisboa, Portugal.*

[3]*Instituto Português de Oncologia de Lisboa Francisco Gentil (IPOLFG), Rua Prof Lima Basto 1099-023 Lisboa, Portugal.*

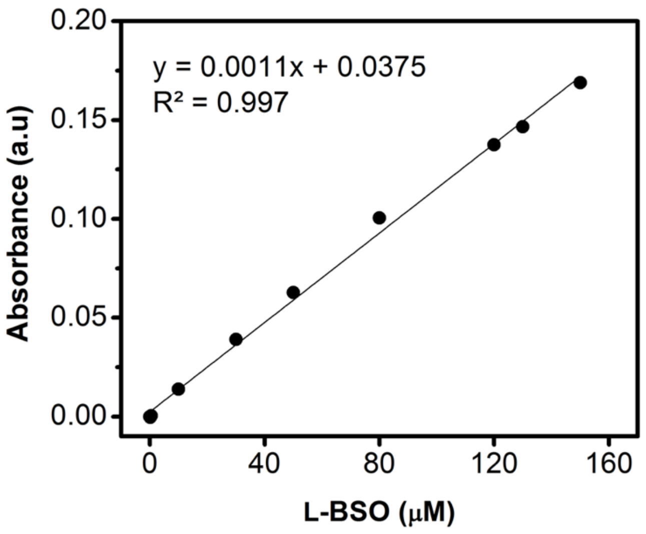

Figure S1. Calibration curve of L-BSO derivatization. Absorption measured at 503 nm.

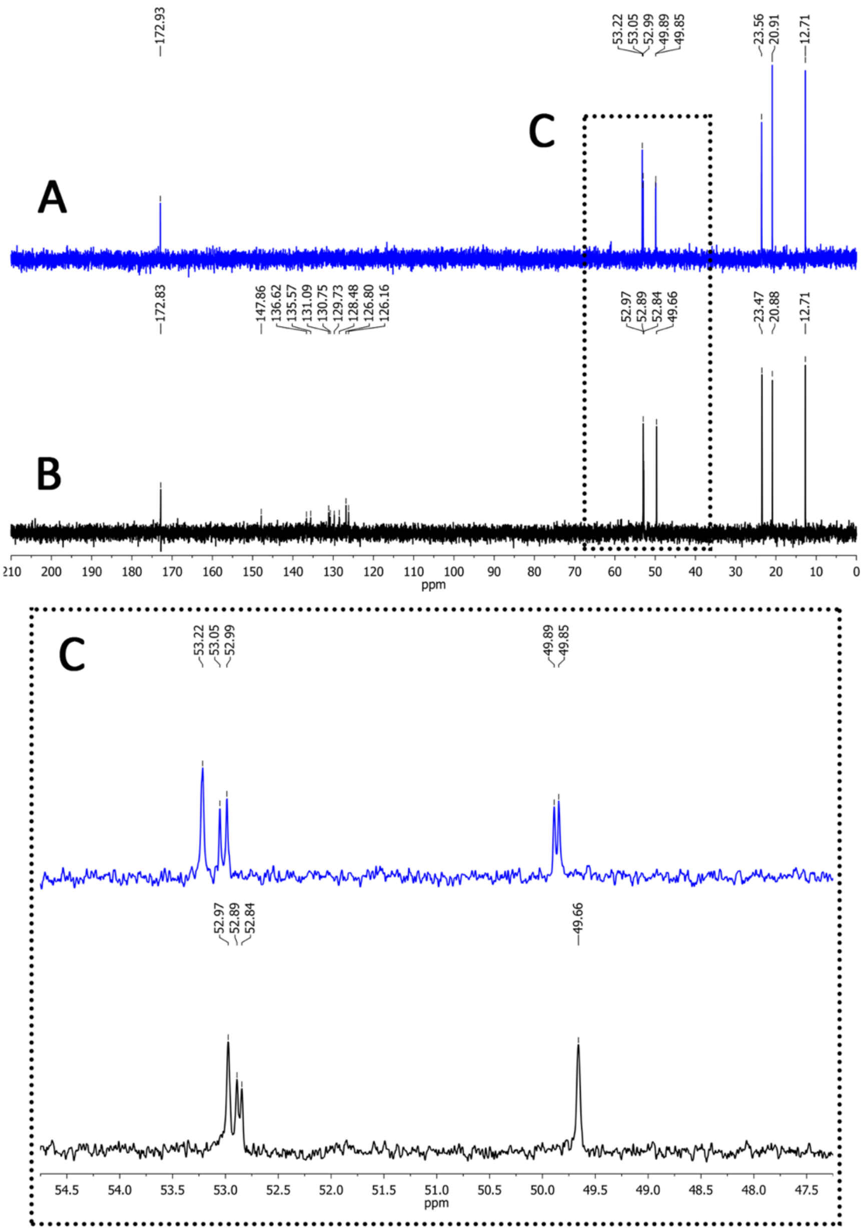

Figure S2. ^{13}C NMR spectra of L-BSO *in situ* derivatization reaction followed by NMR. Comparative spectrum of (A) L-BSO and (B) L-BSO derivative (**3**). Amplification of the spectra in the region 49-45 ppm (C) is also shown.
